# How does livelihood capital alleviate psychological anxiety among immigrants?

**DOI:** 10.3389/fpubh.2025.1700964

**Published:** 2025-11-12

**Authors:** Huishuang Jin, Hui Liu

**Affiliations:** School of Economics, Hunan Agricultural University, Changsha, China

**Keywords:** anxiety, livelihood capital, community governance capacity, strategies, migration, China

## Abstract

The world is facing a surge in migration, with global migrant numbers soaring. Their mental health issues cannot be overlooked. Livelihood capital, as a core resource for human survival and development, plays a vital role in safeguarding immigrants’ living standards and alleviating anxiety. Existing research has focused on single dimensions such as education, social capital, and housing conditions, overlooking the fact that livelihood capital is the result of the synergistic effects of multiple forms of capital. Moreover, previous literature has overlooked the role played by communities. Exploring this issue from the perspective of community governance capacity can fill this gap. Therefore, this study employs micro-level survey data from Hunan Province, China, and utilizes models such as the Oprobit model to empirically analyze the impact of livelihood capital on the anxiety levels of relocated poverty alleviation migrants. It also examines the roles played by livelihood strategies and community governance capabilities in this process. The regression results indicate that livelihood capital significantly reduces immigrants’ anxiety levels. Mechanism testing results indicate that livelihood capital can alleviate migrants’ psychological anxiety by improving livelihood strategies. Community governance capacity exerts a positive moderating effect on the influence of livelihood capital on immigrants’ psychological anxiety. Moreover, the contribution of livelihood capital to immigrants’ psychological anxiety varies across different settlement patterns, relocation duration, and age structures. Therefore, efforts should be made to enhance immigrants’ livelihood resources through multiple channels, strengthen the alignment between livelihood resources and livelihood strategies, and improve community governance capabilities, thereby safeguarding immigrants’ mental health.

## 1 Introduction

Anxiety is a negative emotion (1), and it increases the risk of developing mental health issues. This unhealthy mindset is more likely to emerge among immigrant communities. They transformed identity, culture, and climatic conditions, as their living environment shifted from the familiar to the unfamiliar (2). However, this process takes a long time and is often accompanied by uncertainty about the unknown future and livelihood concerns, ultimately leading to psychological anxiety. In recent years, the global food crisis has continued to worsen, constrained by factors such as extreme climate change, geopolitical conflicts, biofuel production, and economic recession pressures. In 2024, the number of people displaced by food crises reached 99.1 million, doubling from 2013 levels. Displacement increases the likelihood of migration-related anxiety (3), with these refugees and migrants being more prone to mental health issues such as depression and suicidal tendencies than native populations. Psychological distress and apprehension about social integration become significant factors affecting their well-being and sense of belonging.

Mental health issues among immigrant communities have garnered widespread attention from all sectors. Existing literature predominantly analyzes immigrant anxiety from multiple perspectives, including the policy context of immigrant families (4), types of ancestry (5), regional immigration policies (6), immigration status (7), adolescent groups (8), and migration motives (9). A small number of scholars have recognized that livelihood capital—such as education and housing—is a significant factor influencing anxiety, yet they overlook the holistic nature of livelihood capital. Research has found that gender and reputation mechanisms influence the likelihood of anxiety symptoms occurring, and stigmatization exacerbates this negative effect (10). Academic performance, living conditions, and other factors influence students’ mental health levels, with those residing in their own properties experiencing lower anxiety levels (11). Economic crises and recessions are detrimental to adults’ healthy development. As economic welfare improves, anxiety levels decrease significantly (12). Social support negatively influences social anxiety (13), and credit-related social capital mediates the relationship between adverse childhood experiences and anxiety in adulthood (14). Social capital may also stimulate anxiety, thereby influencing suicidal behavior (15).

As the core resource for immigrants’ survival and development, livelihood capital offers new avenues for alleviating psychological anxiety through its diversified accumulation and optimized allocation. Among existing studies on rural household livelihoods, the most widely applied framework is Sustainable Livelihoods Approach proposed by the Department for International Development. This framework posits that livelihood capital is crucial (16). Farmers are both the subjects of survival and the agents of action within vulnerable contexts, where the stock of their livelihood capital and its allocation patterns influence outcomes (17). Livelihood strategies serve as the bridge connecting livelihood capital to livelihood outcomes (18). Households with differing capital stocks mitigate anxiety stemming from economic pressures by shaping differentiated livelihood strategies.

China’s relocation-based poverty alleviation program represents a unique form of population migration, serving as a government-led, top-down poverty reduction initiative. This policy aims to break the constraints imposed by geographical location on economic development by relocating impoverished rural populations living in areas with harsh production conditions, fragile ecosystems, and frequent natural disasters to centralized settlements. Beyond the traditional push factors driving migration from origin areas—such as employment, education, and healthcare—and the pull factors in destination areas—including job opportunities, wages, and educational standards (19)—additional forces emerge between migration sources and destinations through government policies and community dynamics. The quality of community governance profoundly influences migrants’ recovery and development. This approach not only achieves multiple objectives of poverty alleviation, ecological improvement, and social development (20), but also breaks the vicious cycle between human activities and the ecological environment. Although poverty alleviation relocation has positive effects on impoverished populations in poverty-stricken areas, the socioeconomic development of resettlement sites, and ecological restoration in relocation areas, relocated residents also face the risk of falling into a “relocation trap”. Their production systems and social networks have been disrupted, and the loss and insufficiency of these livelihood resources directly trigger anxiety among migrants.

Given this, this paper comprehensively measures the livelihood capital levels of migrants based on survey data from poverty alleviation relocation programs in Hunan Province, China. From the perspectives of livelihood strategies and community governance capacity, this study examines the impact of livelihood capital on psychological anxiety among immigrants and its underlying mechanisms. This paper may contribute in the following three ways: First, it focuses on examining the impact of livelihood capital on immigrants’ psychological anxiety and attempts to explore the underlying mechanisms from the perspectives of livelihood strategies and community governance capacity, offering a new perspective for understanding immigrants’ mental health issues. Second, we analyze whether the impact of livelihood capital on migrants’ psychological anxiety varies across characteristics such as resettlement patterns, relocation duration, and age structure, thereby contributing to our understanding of the heterogeneous effects of livelihood capital and psychological anxiety. Third, using relocated poverty-stricken populations as research subjects helps provide empirical evidence for other countries and populations striving to overcome poverty through relocation.

The remainder of this paper is organized as follows: Section 2 examines the impact of livelihood capital on psychological anxiety among migrants and proposes three hypotheses. Section 3 outlines the data and methodology. Section 4 discusses the evaluation results. Section 5 summarizes the key findings and policy implications.

## 2 Theoretical analysis

### 2.1 Direct impact of livelihood capital on psychological anxiety

Livelihood capital serves as the core resource for farmers to sustain their livelihoods, primarily encompassing five key aspects. Among these, natural capital constitutes an indispensable resource for farmers engaged in agricultural operations (21), being intrinsically linked to both their production activities and daily lives. The higher the natural resource endowment possessed by farmers, the greater their profits from agricultural production activities. This makes farming households, whose primary income comes from agriculture, highly dependent on natural resources. Immigration relocation has significantly reduced the income levels of this group (22). The lack of transitional support mechanisms during livelihood conversion increases the difficulty of adapting to new livelihoods, making individuals more prone to psychological anxiety. Physical capital refers to the infrastructure that farming households rely on to sustain their production and daily lives. Relocation has improved the production conditions and living environment for resettled residents, significantly enhancing their physical capital. The establishment of comprehensive supporting facilities and a living support network in resettlement areas facilitates the social integration of farmers and enhances the adaptability of migrants (23). Human capital is the total of resources people possess, including labor, knowledge, skills, and health. The quantity and quality of human capital directly influence immigrants’ production and lifestyle, and possessing higher human capital facilitates immigrants’ transition toward non-agricultural livelihoods. Social capital embodies the production systems and social networks of farming households (24), with households possessing higher levels of social capital having greater access to social resources. Financial capital serves as the foundation of farmers’ livelihoods, primarily encompassing their deposits, loans, and other financial assets (25). Enhancing material assets, accumulating financial savings, and strengthening farmers’ social capital and networks—these measures all contribute to improving migrants’ adaptability and reducing their anxiety. Based on the above analysis, we propose the following hypothesis:

*Hypothesis 1*: Livelihood capital directly influences immigrants’ psychological anxiety.

### 2.2 The mediating role of livelihood strategies

Livelihood strategies are action plans adopted by farming households to achieve their livelihood goals. Livelihood capital not only directly impacts immigrants’ mental health but also exerts indirect effects through livelihood strategies. Different stocks and allocations of livelihood capital lead immigrants to adopt distinct livelihood strategy types, resulting in varying psychological states among immigrants with different livelihood strategy types. In environments where livelihood risks arise from natural disasters, policy interventions, and other factors, the transformation of farmers’ livelihood strategies primarily stems from the interplay between policies and institutions and livelihood capital. Policies and institutions compel farmers to voluntarily or involuntarily alter their subsistence livelihoods, leading to certain consequences (26). China’s poverty alleviation relocation policy operates on the same principle. Households compare past strategies against existing ones, forecast future developments, and subsequently adjust their livelihood approaches to ultimately select strategies that achieve their livelihood goals. Moreover, there are significant differences in resource allocation among farming households at different stages of livelihood transformation (21). By building up reserves of multidimensional capital—including material, social, and human capital—migrants can not only improve their livelihood standards (27) and bolster their confidence in development, but also shield themselves from psychological anxiety and distress associated with migration (28). For instance, households with higher levels of human capital and social capital tend to be better informed and more adaptable in their livelihoods, enabling them to adjust to changing environments and reduce anxiety stemming from economic pressures and social integration challenges. Based on the above analysis, we propose the following hypothesis:

*Hypothesis 2*: Livelihood capital influences immigrants’ psychological anxiety through livelihood strategies, with livelihood strategies serving as mediators.

### 2.3 The moderating role of community governance capacity

Community governance capacity, as a key environmental factor for immigrants’ mental health, has seen its underlying mechanisms gradually emerge as a cutting-edge topic in the field of immigrants’ emotional integration. Immigrants relocating to new communities lose their familiar surroundings and social networks, which can undermine their self-concept and self-regulation abilities (29). This will lead to immigrants facing loneliness and confusion, exacerbating their anxiety. Community governance provides an opportunity to bridge emotional and social divides. Communities serve as the link between immigrants and their new settlements. Effective governance can foster interaction between immigrants and native residents, connecting immigrants and local communities through organized activities and participatory decision-making. This approach promotes a sense of belonging while reducing the anxiety and unease immigrants experience in their new environment (30). On the one hand, efficient community governance can accelerate the conversion and utilization of immigrants’ livelihood resources, amplifying their effect in alleviating feelings of anxiety. On the other hand, enhancing community governance capabilities can accelerate immigrants’ sense of identification and belonging toward their new communities, reduce external pressures such as cultural exclusion, and reinforce the anxiety-alleviating effects of livelihood capital. For instance, human capital serves as a vital resource for enhancing immigrants’ employment competitiveness. Efficient community governance can organize job training programs, arrange employment opportunities, and strengthen social capital connections, thereby alleviating anxiety stemming from income uncertainty and social disconnection. In summary, we propose the following hypothesis:

*Hypothesis 3*: Community governance capacity moderates the effect of livelihood capital on psychological anxiety.

**Figure 1:**
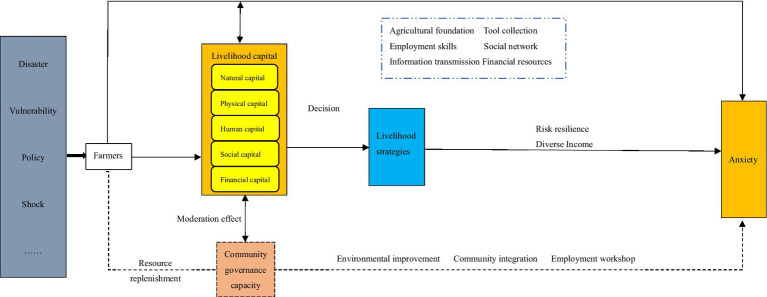
Analytical framework.

The analysis framework is depicted in Figure 1.

## 3 Research design

### 3.1 Data sources

Hunan Province pioneered China’s targeted poverty alleviation policy and serves as a model in poverty eradication practices. The data used in this study were collected from a 2020 survey in County F, Hunan Province. County F, situated on the western edge of Hunan Province, features complex terrain and is a typical deeply impoverished area. It also serves as a primary relocation site for Hunan’s poverty alleviation efforts, making it highly representative. Micro-level research employed random sampling for interviews. After data verification and cleaning, 496 valid samples were ultimately obtained.

### 3.2 Variable specifications

#### 3.2.1 Dependent variable

Psychological anxiety level is the dependent variable in this study. Anxiety, as an emotion experienced by immigrants, serves as a crucial subjective indicator for assessing mental health. Therefore, the questionnaire item “Since relocating, how anxious have you felt?” was selected for measurement. Respondents were given five response options: Very anxious, Somewhat anxious, Neutral, Slightly anxious, and Not anxious, assigned numerical values from 1 to 5, respectively.

#### 3.2.2 Core explanatory variables

Livelihood capital, as the core foundation for immigrants to sustain their livelihoods and achieve development, plays a vital role not only in providing material security but also in alleviating psychological anxiety. Livelihood capital primarily encompasses five dimensions: natural, material, social, human, and financial capital (31, 32), employing an entropy-weighted-TOPSIS integrated measurement approach. Among these, physical capital is primarily measured by the quantity of household production and living tools, productive infrastructure, and satisfaction with current production methods; human capital is measured by educational attainment, labor force size, and health status; social capital is measured using participation in public affairs, neighborly trust, and communication network costs; financial capital is measured by borrowing and income; additionally, natural capital is proxied by the amount of land under cultivation (Table 1).

**Table 1:** Livelihood capital indicator system.

Variable categories	Variable name	Assignment
Physical capital	Number of household production and living tools	Continuous variable
	Level of development of productive infrastructure	1 = Very inadequate, 2 = Somewhat inadequate, 3 = Average, 4 = Somewhat adequate, 5 = Very adequate
	Level of satisfaction with the current production methods	1 = Very dissatisfied, 2 = Somewhat dissatisfied, 3 = Neutral, 4 = Somewhat satisfied, 5 = Very satisfied
Human capital	Highest educational attainment among family members	1 = Elementary school and below 2 = Junior high school 3 = High school (vocational school) 4 = College or undergraduate degree 5 = Graduate degree
	Labor force size	1 = 0 people, 2 = 1 person, 3 = 2–3 people, 4 = 3 or more people
	Health status	1 = Very unhealthy, 2 = Fairly unhealthy, 3 = Average, 4 = Fairly healthy, 5 = Very healthy
Social capital	Level of participation in community affairs	1 = Very few, 2 = Few, 3 = Average, 4 = Fairly many, 5 = Very many
	Level of trust in neighbors	1 = Very distrustful, 2 = Somewhat distrustful, 3 = Neutral, 4 = Somewhat trusting, 5 = Very trusting
	Monthly household internet service fees	1 = 100 yuan or less, 2 = 100 to 200 yuan, 3 = 200 yuan or more
Financial capital	The ease of borrowing money from friends and family	1 = Very difficult, 2 = Quite difficult, 3 = Moderately difficult, 4 = Quite easy, 5 = Very easy
	Changes in income	1 = Significant reduction 2 = Some reduction 3 = Essentially unchanged 4 = Some improvement 5 = Substantial improvement
Natural capital	Area of land under management	mu

#### 3.2.3 Mechanism variables

This study examines the potential pathways through which livelihood capital may influence psychological anxiety among immigrants, with livelihood strategies serving as mediating variables. Drawing on relevant research (33), we classify households based on their participation in livelihood activities. The diversity of livelihood strategies is measured by the number of actual livelihood activities undertaken by migrant families. If a single household engages in both agricultural production and migrant work, its livelihood strategy diversity value is 2, and so on. The moderating variable is community governance capacity. Drawing upon existing research findings (2), we employ an entropy-weighted-TOPSIS integrated approach to measure community governance capacity across four dimensions: community staff, leadership, public security levels, and support policies.

#### 3.2.4 Control variables

Drawing on existing research (31, 34, 35), the following control variables were specified: at the individual migrant level, gender, age, Mandarin proficiency, and pension insurance were included; at the household level, cadre support, number of training sessions received, and household size were included; at the community level, relocation distance and cultural diversity were included. The specific definitions and descriptive statistics of these variables are presented in Table 2.

**Table 2:** Variable descriptions and descriptive statistics.

Variables	Meaning and assignment	Mean	SD
Anxiety	1 = Extremely anxious, 2 = Fairly anxious, 3 = Neutral, 4 = Slightly anxious, 5 = Not anxious	3.966	1.116
Livelihood capital	Composite index based on entropy-weighted-TOPSIS calculation	0.408	0.120
Gender	1 = Male, 0 = Female	0.675	0.469
Age	year	48.244	15.462
Mandarin proficiency	1 = Very poor, 2 = Poor, 3 = Average, 4 = Fairly good, 5 = Very good	3.069	1.147
Pension insurance	Whether to participate in pension insurance? 0 = No,1 = Yes	1.181	0.386
Cadre support	Do you have any relatives or friends who are poverty alleviation cadres? 0 = No,1 = Yes	1.131	0.451
Number of training sessions attended	1 = 0 types, 2 = 1 type, 3 = 2 types, 4 = 3 types	1.345	1.015
Population size	Number of household members	5.129	2.033
Relocation distance	Distance between the destination and origin locations	1.228	0.495
Cultural diversity	The ease or difficulty of communicating with indigenous peoples. 1 = Very difficult, 2 = Quite difficult, 3 = Moderately difficult, 4 = Quite easy, 5 = Very easy	4.284	0.820
Community governance capacity	Composite index based on entropy-weighted-TOPSIS calculation	0.747	0.156
Livelihood strategy	Number of livelihood approaches	1.768	0.620

### 3.3 Model specification

#### 3.3.1 Oprobit model

Farmers’ psychological anxiety level is an ordinal variable. Therefore, this study adopts the Oprobit model for estimation, referencing relevant research (36). The specific model specification is as follows:


$$Y_{i}^{*}=\alpha_{1}X_{i}+\alpha_{2}Z_{i}+\varepsilon_{i}$$ (1)

$$Y_{i}=\{\begin{array}{l} 1,\text{if}\;Y_{i}^{*}\leq \gamma_{1}\\ 2,\text{if}\;\gamma_{1}<Y_{i}^{*}\leq \gamma_{2}\\ 3,\text{if}\;\gamma_{2}<Y_{i}^{*}\leq \gamma_{3}\\ 4,\text{if}\;\gamma_{3}<Y_{i}^{*}\leq \gamma_{4}\\ 5,\text{if}\;Y_{i}^{*}>\gamma_{4} \end{array}$$ (2)

In Equations 1, 2, $Y_{i}^{*}$ represents the latent variable for farmers’ psychological anxiety levels; *X*_i_ denotes livelihood capital variables; *Z*_i_ represents control variables; $\gamma_{1}$ , $\gamma_{2}$ , $\gamma_{3}$ , $\gamma_{4}$ denote the unknown cut-off points for the psychological anxiety variable, with $\gamma_{1}$ < $\gamma_{2}$ < $\gamma_{3}$ < $\gamma_{4}$ ; *α*₁ and α₂ denote the estimated coefficients; $\varepsilon_{i}$ denotes the error term.

#### 3.3.2 Mechanism verification

Furthermore, to examine the mechanism through which livelihood capital influences psychological anxiety, the following model is constructed:


$$M_{i}=\rho_{0}+\rho_{1}X_{i}+\rho_{2}Z_{i}+\varepsilon_{1i}$$ (3)

$$Y_{i}=\rho_{3}+\rho_{4}X_{i}+\rho_{5}M_{i}+\rho_{6}Z_{i}+\varepsilon_{2i}$$ (4)

$$Y_{i}=\alpha_{3}X_{i}+\alpha_{4}X_{i}\times D_{i}+\alpha_{5}D_{i}+\alpha_{6}Z_{i}+\varepsilon_{3i}$$ (5)

In Equations 3–5, $M_{i}$ and $D_{i}$ represent the livelihood strategy of the mediating variable and the community governance capacity of the moderating variable, respectively; *X*_i_ denotes the production capital variable; *X*_i_ × *D*_i_ represents the interaction term between livelihood capital and community governance capacity. To avoid potential multicollinearity arising from interaction terms, the model employs centering treatment for these interaction terms.

## 4 Results analysis

### 4.1 Collinearity test

Considering the potential issue of multicollinearity among different explanatory variables, the variance inflation factor (VIF) was employed to assess multicollinearity. Results indicate that the maximum VIF value among the explanatory variables in the model is 1.81, the minimum is 1.04, and the mean is 1.30. All values are significantly below the critical threshold of 10, indicating no severe multicollinearity among variables in the model. This enhances the reliability of the estimation results.

### 4.2 Benchmark regression

Table 3 reports the benchmark regression results for the impact of livelihood capital on psychological anxiety. Column (1) presents the model without controlling variables, while columns (2) to (4) progressively incorporate individual immigrant characteristics, household characteristics, and community characteristics based on Column (1). Column (5) presents the model incorporating livelihood capital variables across dimensions. As shown in Table 3, livelihood capital exerts a positive influence on psychological anxiety, which is statistically significant at the 1% level. This indicates that an increase in livelihood capital can reduce immigrants’ anxiety levels. Furthermore, Column (5) indicates that human capital and natural capital positively influence immigrants’ anxiety at the 1% significance level, while material capital negatively affects psychological anxiety at the 10% significance level. The effects of social capital and financial capital on anxiety are not statistically significant.

**Table 3:** Benchmark regression results.

Variables	(1)	(2)	(3)	(4)	(5)
Livelihood capital	2.574***	2.689***	3.802***	3.437***	
	(0.428)	(0.458)	(0.610)	(0.638)	
Physical capital					−1.092*
					(0.648)
Human capital					1.589***
					(0.477)
Social capital					−0.090
					(0.215)
Financial capital					0.441
					(0.350)
Natural capital					1.728***
					(0.333)
Gender		−0.035	0.003	0.046	0.066
		(0.114)	(0.117)	(0.118)	(0.121)
Age		0.011***	0.009**	0.007*	0.006
		(0.004)	(0.004)	(0.004)	(0.004)
Mandarin proficiency		0.080	0.070	0.007	0.027
		(0.055)	(0.057)	(0.059)	(0.060)
Pension insurance		0.131	0.113	0.226	0.324**
		(0.146)	(0.145)	(0.155)	(0.152)
Cadre support			−0.285*	−0.287*	−0.232
			(0.147)	(0.149)	(0.148)
Number of training sessions attended			−0.257***	−0.155**	−0.203***
			(0.068)	(0.071)	(0.076)
Population size			−0.014	−0.021	0.013
			(0.027)	(0.028)	(0.029)
Relocation distance				−0.136	−0.085
				(0.096)	(0.102)
Cultural diversity				0.387***	0.360***
				(0.066)	(0.069)
Wald chi2	36.11	49.53	63.00	98.21	116.50
R2	0.0284	0.0357	0.0528	0.0820	0.1123
N	496	496	496	496	496

***, **, and * denote significance levels of 1, 5, and 10%, respectively, with robust standard errors indicated in parentheses.

### 4.3 Robustness test

Robustness tests were conducted using substitution models, redefining explanatory variables, and instrumental variable method. First, we re-estimated the model using multiple linear regression. Column (1) of Table 4 reports the estimation results. Findings indicate that livelihood capital exerts a significant positive effect on psychological anxiety, showing no substantive deviation from the benchmark regression results. Second, we employed an ordered logit model for estimation. As shown in Column (2) of Table 4, livelihood capital continues to exert a significant positive influence on immigrants’ anxiety levels. Third, we remeasured livelihood capital levels using principal component analysis. Table 4 column (3) reports the regression results for this alternative measure. The findings show that after remeasuring the explanatory variables, the estimation results remain consistent with the core conclusions presented earlier, indicating the robustness of this study’s findings. Finally, to address potential endogeneity issues, this paper adopts the distance of communities from towns as an instrumental variable (IV), following existing research (36). Then, employs the Conditional Mixed Process (CMP) estimation method to address endogeneity issues. The estimation results indicate that atanhrho_12 is significantly different from 0 at the 1% level, suggesting that livelihood capital exhibits endogeneity in the estimated model. This also confirms the validity of employing the CMP model to address endogeneity. After endogeneity control, livelihood capital is significantly positive at the 1% statistical level, showing no substantive difference from the benchmark regression results (Table 4).

**Table 4:** Robustness test estimation results.

Variables	(1) OLS	(2) Ordered logit	(3) Redefining explanatory variables	(4) CMP
Livelihood capital	3.005***	5.609***	0.817***	10.757***
	(0.548)	(1.200)	(0.214)	(0.828)
IV				0.025***
				(0.010)
Atanhrho_12				−1.184***
				(0.398)
Control variables	YES	YES	YES	YES
Wald chi2/*F*	16.65	107.09	69.75	1680.49
*R* ^2^	0.1963	0.0932	0.0950	
*N*	496	496	496	496

*** denotes a 1% significance level; values in parentheses represent robust standard errors.

### 4.4 Mechanism verification

As demonstrated earlier, livelihood capital influences migrants’ mental health through livelihood strategies, and its effect on migrants’ anxiety is further moderated by community governance capacity. First, to further validate the mediating role of livelihood strategies, this study incorporates the livelihood strategy variable into the analytical framework and constructs a mediation effect model for testing, with results presented in Table 5. Column (1) indicates that livelihood capital significantly and positively influences livelihood strategies. This implies that households with more abundant livelihood capital possess greater capacity and choice to adopt diverse livelihood approaches, thereby reducing the risk of reliance on a single income source and enhancing migrants’ well-being. Column (2) shows that both livelihood capital and livelihood strategies positively affect psychological anxiety, with significance at the 1 and 5% statistical levels, respectively. A possible explanation is that after relocation, migrants experience improved living conditions. Government-organized employment skills training and job referrals ensure basic survival for relocated households, naturally reducing anxiety. Meanwhile, the diversity of livelihood approaches provides migrants with clear goals for increasing income, alleviating feelings of uncertainty about the future, and thereby easing anxiety.

**Table 5:** Mechanism testing.

Variables	(1) Livelihood strategy	(2) Psychological anxiety	(3) Psychological anxiety
Livelihood capital	1.331**	3.304***	2.784***
	(0.591)	(0.647)	(0.784)
Livelihood Strategy		0.209**	
		(0.095)	
Community governance capacity			3.600***
			(0.512)
Livelihood capital × Community governance capacity			7.148**
			(3.336)
Control variables	YES	YES	YES
Wald chi^2^	43.07	108.85	156.31
*R* ^2^	0.0494	0.0865	0.1390
*N*	496	496	496

*** and ** denote significance levels of 1 and 5%, respectively, with robust standard errors indicated in parentheses.

To further validate the moderating role of community governance capacity, this study incorporates community governance capacity into the framework and constructs a moderation effect model for testing, with results shown in Column (3) of Table 5. Column (3) indicates that community governance capacity exerts a significant positive influence on psychological anxiety and positively moderates the promoting effect of livelihood capital on psychological anxiety. A possible reason is that well-governed resettlement areas provide supporting educational and medical resources, thereby reducing challenges such as accessing healthcare and schooling for migrants. Some communities employ counselors and regularly organize cultural activities, which can alleviate residents’ loneliness, directly reducing anxiety through psychological adaptation and livelihood security. Large resettlement areas also feature robust employment mechanisms that connect job training with job opportunities, helping migrants convert livelihood capital into income sources and ultimately enhancing livelihood capital’s anxiety-reducing effects.

### 4.5 Heterogeneity analysis

The impact of livelihood capital on psychological anxiety may exhibit certain heterogeneity. Therefore, this study conducted heterogeneity tests based on three grouping criteria: whether the resettlement model involved land-based centralized relocation, whether the relocation duration exceeded 1 year, and household age structure. The estimation results are presented in Tables 6, 7.

**Table 6:** Heterogeneity estimates based on resettlement patterns and relocation duration.

Variables	Resettlement patterns		Relocation duration	
	(1) Yes	(2) No	(3) Yes	(4) No
Livelihood capital	4.979***	3.656***	4.635***	3.766***
	(0.960)	(0.887)	(0.873)	(1.217)
Control variables	YES	YES	YES	YES
Wald chi^2^	43.07	67.57	68.93	29.99
*R* ^2^	0.0936	0.0926	0.1143	0.0675
*N*	231	265	276	220

*** denotes a 1% significance level; values in parentheses represent robust standard errors.

**Table 7:** Heterogeneity estimates based on age structure.

Variables	(1) Child-rearing and older adults care households	(2) Child-rearing households	(3) Older adults care households	(4) Burden-free households
Livelihood capital	2.913***	2.609	6.139***	7.242***
	(0.906)	(1.593)	(1.674)	(1.654)
Control variables	YES	YES	YES	YES
Wald chi^2^	40.80	23.74	52.35	40.91
*R* ^2^	0.0618	0.1066	0.1737	0.1921
*N*	215	104	100	77

*** denotes a 1% significance level; values in parentheses represent robust standard errors.

The regression results in columns (1) and (2) of Table 6 indicate that livelihood capital significantly alleviates anxiety regardless of whether the resettlement model involves land allocation. However, the estimated coefficients for livelihood capital reveal that the coefficient for the land-allocation resettlement group is markedly higher than that for the non-land-allocation resettlement group. One possible reason is that the centralized resettlement model typically relocates residents from the same village to a single area. This approach not only provides better housing for migrants but also fosters tighter social networks, effectively reducing their sense of disorientation in unfamiliar surroundings. Simultaneously, the allocation of farmland directly ensures migrants’ livelihoods, enabling them to engage in normal production and daily life—a key factor in alleviating anxiety.

The regression results in columns (3) and (4) of Table 6 indicate that livelihood capital significantly alleviates psychological anxiety among migrants in both sample groups—those with relocation durations exceeding 1 year and those with durations of 1 year or less. Moreover, the estimated coefficient is larger in the group with durations exceeding 1 year. This suggests that relocation duration is a crucial factor in migrants’ adaptation to new lives. As time accumulates, migrants’ livelihood capital gradually aligns with the resettlement area, thereby exerting a stronger anxiety-alleviating effect.

This study categorizes age structures into four types based on the characteristics of China’s rural population. First, households with children under 14 and members over 60 are defined as child-rearing and older adults care households. Second, households with children under 14 but no members over 60 are defined as child-rearing households. Third, households without children under 14 but with members over 60 are defined as older adults care households. Finally, households where all members are aged between 14 and 60 are defined as burden-free households.

The regression results in Table 7 indicate that livelihood capital exerts a significant positive influence on psychological anxiety in child-rearing and older adults care households, older adults care households, and burden-free households. Only child-rearing households show no significant effect. Regarding estimated coefficients, burden-free households exhibit the highest coefficient, followed by older adults care households, while child-rearing households have the smallest coefficient. A possible explanation is that burden-free households possess a more effective labor force, enabling livelihood capital to directly impact production and daily life, thereby effectively enhancing migrants’ psychological security. The minimal coefficient and lack of significant effect for child-rearing households may stem from their livelihood capital being partially offset by rigid expenditures such as children’s education and healthcare, with parenting pressures weakening its anxiety-alleviating effects.

### 4.6 Discussion

Farmers’ psychological well-being is closely tied to their household livelihood capital. Human capital and natural capital reduce farmers’ anxiety levels, while material capital negatively impacts anxiety. A possible reason lies in the fact that after relocation, immigrants’ material assets face a “discontinuity” and “reconstruction.” They need to acquire new material assets, and their material capital is difficult to accumulate rapidly in the short term. Meanwhile, the cost of living in resettlement areas exceeds that of their original places of residence, and income growth lags behind rising costs. Consequently, increases in material capital may subject migrants to economic pressures, thereby intensifying their anxiety. Human capital can enhance farmers’ competitiveness in non-agricultural employment, and immigrants with higher human capital levels are more receptive to new technologies. They have broader income sources, which reduces anxiety stemming from livelihood uncertainty. Natural capital serves as the fundamental livelihood source for farming households, with the amount of land they cultivate determining the extent of immigrants’ confidence in the future. Stable land yields provide families with a continuous supply of food and income, not only alleviating basic survival anxieties but also fostering a psychological sense of control over their lives. This instills a mindset that “land brings peace of mind.” This study uses Hunan Province, China, as its sample area and is highly applicable to agricultural regions in central and western China, as well as policy-driven migrant populations. It also holds a certain reference value for some developing countries, particularly for groups migrating from rural to urban areas. Such migrants share common challenges in livelihood capital and sources of anxiety (such as employment pressures). Enhancing livelihood capital can improve income sources and strengthen adaptive capacity.

From the perspective of livelihood strategies, the accumulation of livelihood capital provides multidimensional support for transforming livelihood approaches and creates conditions for expanding livelihood sources. Livelihood strategies serve as a vital link between livelihood capital and psychological well-being, playing a significant role in alleviating anxiety. Diversifying livelihood sources helps mitigate the impact of single-market fluctuations and enhances migrants’ resilience against risks. However, we should also recognize that livelihood strategies have limited potential. The reasons are as follows: First, the realization of livelihood strategy potential is constrained by multiple factors. According to survey data, immigrants possess limited livelihood capital, particularly marked by a significant reduction in natural capital and pronounced deficiencies in human capital. These imbalances make it difficult for immigrants to upgrade their livelihood strategies through capital accumulation. Instead, they may fall into a “low-level equilibrium” trap, thereby failing to effectively alleviate their anxiety. Second, the transformation of immigrants’ livelihood strategies requires time and accumulated experience. However, the average relocation period for the sample immigrants was less than 2 years, leaving them insufficient time to adapt to the transformed production and living environments. Furthermore, the use of cross-sectional data in the study makes it difficult to capture the cumulative effects of livelihood capital accumulation and the transformative processes of livelihood strategies. These gaps and limitations prevent livelihood strategies from fully realizing their substantial catalytic effects.

It is worth noting that our study has certain limitations, and future research can explore further in many aspects. First, this study employs cross-sectional data, which will limit the generalizability of findings to the temporal dimension. Panel data will be utilized to examine long-term dynamic effects. Second, while the study currently focuses on County F in Hunan Province as a representative sample area, future research may gradually expand to multiple counties or even cover several provinces. This expansion will enable a more comprehensive analysis of various factors, including the educational attainment and employment types of relocated households, as well as the development surrounding their resettlement communities. The study also compared the heterogeneous characteristics across different regions. Additionally, we will consider analyzing influencing factors from multiple perspectives, such as identity recognition and social integration, thereby enhancing the scientific rigor and accuracy of the research.

## 5 Conclusion and suggestions

### 5.1 Conclusion

The relocation policy for poverty alleviation is a crucial component of China’s poverty reduction strategy, aiming to lift people out of poverty through resettlement. However, such relocation policies can disrupt migrants’ social networks and drastically alter their living environments, triggering psychological anxiety. Drawing on sustainable livelihoods theory and utilizing survey data from Hunan Province, this study empirically examines the impact of livelihood capital on immigrants’ psychological anxiety. The findings reveal the following insights:

(1) Overall, livelihood capital can alleviate immigrants’ anxiety, but the reinforcing effects and outcomes vary across different dimensions of capital. Human capital and natural capital positively influence immigrants’ anxiety levels, while material capital has the opposite effect. The impact of social capital and financial capital on anxiety is not significant.
(2) Livelihood capital influences migrants’ psychological anxiety through livelihood strategies, while community governance capacity enhances the positive effect of livelihood capital on anxiety, exerting a positive moderating effect.
(3) Heterogeneity analysis revealed that the impact of livelihood capital on anxiety levels exhibited significant differences across resettlement patterns, relocation duration, and age structure. Livelihood capital contributed more substantially to the psychological well-being of migrants resettled in land-based, concentrated communities and those relocated for over 1 year. It significantly alleviated anxiety among families with childcare and older adult care responsibilities, older adult-focused households, and households without dependents, though the degree of significance varied across these groups.

### 5.2 Suggestions

(1) Livelihood capital serves as the cornerstone for enhancing immigrants’ adaptability, necessitating its enhancement through multiple channels. First, when resettling these immigrants, prioritize clustering arrangements based on shared origins, concentrating individuals from the same place of departure in adjacent areas to effectively preserve their existing social networks. Additionally, strive to redistribute land to immigrants to ensure their basic livelihood needs are met. Second, vocational training programs should be established based on local industrial needs, with practical training bases set up within industrial parks. Migrants acquiring vocational skills should be prioritized for placement in partner enterprises to facilitate employment. Third, the government should increase investment in labor-intensive industries, formulate policies to support micro and small enterprises, and encourage capable farmers to start their own businesses. Finally, dedicated credit products should be established for relocated households, offering low-interest or interest-free loans to meet their entrepreneurial needs. Simultaneously, enterprises hiring migrants should receive loans and tax incentives to boost employment rates among the relocated population.

(2) The government should strengthen top-level design and dispatch personnel to conduct in-depth visits and research on the livelihood resources of immigrants. Classify immigrants based on factors such as age, occupation, skill level, material capital, and social networks, then use big data to identify industries suitable for the development of various immigrant groups. In particular, prioritize the development of industries that align with natural resources to reduce investment costs. For instance, migrants in mountainous areas can prioritize developing forest-based economies. The government should spearhead the establishment of a “contract-based training plus full-chain support” mechanism to secure income sources and alleviate economic anxieties. Simultaneously, establish a dynamic database of immigrant livelihood resources, regularly updating key indicators such as age and skill levels to provide data support for precise industry-job matching.

(3) Improving community governance capacity and enhancing immigrants’ sense of community integration. First, we should set up community psychological service stations and set up a ledger to pay close attention to the mental health problems of immigrants. For immigrants with depression, anxiety, and other psychological diseases, regular communication, effective guidance, providing jobs, and other ways can help reduce the economic and social pressure of immigrants. Second, the community should organize cultural activities regularly to guide and encourage farmers to actively participate in community public affairs, such as voting and other activities. Third, we should encourage participatory governance methods such as meritocracy and collective consultation to strengthen migrants’ sense of community identity. It is necessary to make full use of bulletin boards, loudspeakers, WeChat groups, and other publicity means to mobilize the enthusiasm of immigrants to participate, and adopt door-to-door publicity to improve the familiarity of middle-aged and older immigrants with community governance methods, encourage them to actively participate in community autonomy, and expand social networks.

## Data Availability

The raw data supporting the conclusions of this article will be made available by the authors, without undue reservation.

## References

[1] Creswell C Waite P Hudson J. Practitioner review: anxiety disorders in children and young people – assessment and treatment. J Child Psychol Psychiatry. (2020) 61:628–43. doi: 10.1111/jcpp.13186, PMID: 31960440

[2] Shi J Xian Z Zhu T Kang X. Research on livelihood capital, endogenous development momentum and sustainable livelihoods of relocated farmers. Int Rev Econ Finance. (2025) 102:104259-104259. doi: 10.1016/J.IREF.2025.104259

[3] Canady AV. JAMA: displacement raises odds of depression, anxiety. Ment Heal Wkly. (2025) 35:7. doi: 10.1002/mhw.34576

[4] Manalo-Pedro E Enriquez LE Nájera JR Ro A. Anxious activists? Examining immigration policy threat, political engagement, and anxiety among college students with different self/parental immigration statuses. J Health Soc Behav. (2024) 65:381–99. doi: 10.1177/00221465241247541, PMID: 38682706 PMC11380356

[5] Hussen SA Tewodros T Argaw SA Berhanu L Tesfai R Easley JA . Immigration, discrimination, and resilience: intersecting social factors associated with symptoms of depression and anxiety among Ethiopian and Eritrean American emerging adults. J Racial Ethn Health Disparities. (2025). doi: 10.1007/s40615-025-02307-x, PMID: 39982584

[6] Becerra D Hernandez G Porchas F Castillo J Nguyen V Perez G. Immigration policies and mental health: examining the relationship between immigration enforcement and depression, anxiety, and stress among Latino immigrants. J. Ethn Cult Divers Soc Work. (2020) 29:43–59. doi: 10.1080/15313204.2020.1731641

[7] Kurdi V Archambault I. Self-perceptions and engagement in low-socioeconomic elementary school students: the moderating effects of immigration status and anxiety. School Ment Health. (2020) 12:400–16. doi: 10.1007/s12310-020-09360-3

[8] Hagan M Lara J Montanes MC. Childhood adversity, socioemotional functioning and generalized anxiety in young adults from mixed immigration status families. Child Abuse Negl. (2021) 118:105128. doi: 10.1016/j.chiabu.2021.105128, PMID: 34051486

[9] Duque M Vos S García M Brown E Perrino T Trujillo J . The importance of motivations for emigration in understanding post-migration mental health outcomes among diasporic Venezuelans in the United States and Colombia. J Cross-Cult Psychol. (2025) 56:663–79. doi: 10.1177/00220221251327966

[10] Wendt WG Chaves WL Costa BA. Exploring the influence of age, gender, stigma, and years living with HIV on mental health outcomes. HIV Med. (2025) 1–10. doi: 10.1111/hiv.70098 PMC12579887 40827545

[11] Nosè M Turrini G Muriago G Badino M Cristofalo D Sartori R . Psychological distress and well-being in university and high school students: a cross-sectional study in Italy. BMJ Open. (2025) 15:e101446. doi: 10.1136/BMJOPEN-2025-101446, PMID: 40829822 PMC12366582

[12] El Murr P Rahme E Younes RS Assaf R Zalaket N. Mental health during the Lebanese economic crisis: association between financial well-being, anxiety, and depression. Int J Soc Psychiatry. (2025):00207640251359059. doi: 10.1177/00207640251359059, PMID: 40819266 PMC12946224

[13] Deng Y Wang X. The impact of physical activity on social anxiety among college students: the chain mediating effect of social support and psychological capital. Front Psychol. (2024) 15:1406452. doi: 10.3389/FPSYG.2024.1406452, PMID: 38957885 PMC11217649

[14] Goodman ML Baker L Maigallo AK Elliott A Keiser P Raimer-Goodman L. Adverse childhood experiences, adult anxiety and social capital among women in rural Kenya. J Anxiety Disord. (2022) 91:102614. doi: 10.1016/J.JANXDIS.2022.102614, PMID: 35988441 PMC11925039

[15] Yu B Chen X. Relationship among social capital, employment uncertainty, anxiety, and suicidal behaviors: a chained multi-mediator mediation modeling analysis. Arch Suicide Res. (2022) 26:261–79. doi: 10.1080/13811118.2020.1793044, PMID: 32697144 PMC7855900

[16] Zhang B Mao W Hu Z Cai Y Xie N Weng Z. How does livelihood capital influence the green production behaviors among professional grain farmers cultivating high-quality rice? Front Nutr. (2025) 12:1555488–8. doi: 10.3389/FNUT.2025.1555488, PMID: 40491587 PMC12146195

[17] Cntexont V Sustainable livelihoods guidance sheets London: Department for International Development (2000) 68–125

[18] Cui H Wang Y Wang W Liu C. Rural households' livelihood transitions in China: processes, drivers and outcomes. China Agric Econ Rev. (2025) 17:171–90. doi: 10.1108/CAER-11-2023-0346

[19] Yudhistira S Nailan N Saefihim S Ratna I Andre Y Afri A. Push-pull theory of migration impact on students decisions at East Java Province. SHS Web Confer. (2025) 212:4025. doi: 10.1051/SHSCONF/202521204025

[20] Liu W Xu J Li J. Livelihood adaptive capacity of rural households under poverty alleviation relocation: a case study of southern Shaanxi. Chin J Agric Resour Reg Plann. (2018) 39:218–23. doi: 10.7621/cjarrp.1005-9121.20181229

[21] Zhang J Fan Z Liu J Ahmad F Cao Z. Livelihood capital, risk response, and rural household poverty vulnerability: an empirical experience based in China. Rev Dev Econ. (2024) 29:1693–711. doi: 10.1111/RODE.13184

[22] Ma M Chen S Tao S. Poverty reduction effect and livelihood development of poverty alleviation relocation in ethnic minority areas of Nujiang prefecture, Yunnan province. J Arid Land Resour Environ. (2021) 35:16–23. doi: 10.13448/j.cnki.jalre.2021.264

[23] Zhang H Shi M. A study on the social adaptation of female migrants in poverty alleviation in inhospitable areas—on the female migration survey of different resettlement modes in the 13th five-year plan period in Ningxia. Ningxia Soc Sci. (2021) 3:163–78. Available online at: https://kns.cnki.net/kcms2/article/abstract?v=6VlBgy5--500egmRfLtaK5cS6W6ADsxXWM508GUGLR90q4x7H8b9aZHlC8iud35JrJ3159yNcO-XB3584lk2LpudmoLkIkAqQBL37raZ04zGSOLo8Lv5WggNgZqAvkvroQTEbPJGRQdp-ikKRI9_I_4XhByBHpVnNNlBXyRB8PXy2zXCyAN5Lg==&uniplatform=NZKPT&language=CHS

[24] Kopytko N. What role can a livelihood strategy play in addressing climate change? Lessons in improving social capital from an agricultural cooperative in Ukraine. Clim Dev. (2018) 10:717–28. doi: 10.1080/17565529.2018.1442787

[25] Chen C Chen R Wang Q Zhang M Song J Zuo W . Deciphering the mechanism of women’s mental health: a perspective of urban–rural differences. Front Public Health. (2025) 13:1545640–11. doi: 10.3389/fpubh.2025.1545640, PMID: 40109412 PMC11921888

[26] Li J Li S Daily GC. Research on rural household livelihood and environmental sustainable Development. Beijing: Social Sciences Academic Press (2017). 23 p.

[27] Chen S Ma M Tao S. The influence of livelihood capital, livelihood strategies and livelihood choice behavior of poverty alleviation relocation from the perspective of common prosperity. J Hohai Univ. (2023) 25:94–108. doi: 10.3876/j.issn.1671-4970.2023.01.010

[28] Iwasaki K Sawada Y Aldrich DP. Social capital as a shield against anxiety among displaced residents from Fukushima. Nat Hazards. (2017) 89:405–21. doi: 10.1007/s11069-017-2971-7

[29] Rodriguez MI Dobler V. Survivors of hell: resilience amongst unaccompanied minor refugees and implications for treatment-a narrative review. J Child Adolesc Trauma. (2021) 14:1–11. doi: 10.1007/S40653-021-00385-7 34820043 PMC8586295

[30] Lu W Zhang W. Emotional integration, governance capacity, and poverty alleviation in relocated communities: a case study of relocated migrants in China. J Knowl Econ. (2024) 16:1–28. doi: 10.1007/S13132-024-01804-4

[31] He Y Li W. The impact of livelihood capital on sustainable livelihood capacity of farmers: evidence from Yunnan Province, China. Appl Econ Lett. (2025) 32:2077–82. doi: 10.1080/13504851.2024.2332536

[32] Wu J Zhang J Yang H. Impact of relocation in response to climate change on farmers’ livelihood capital in minority areas: a case study of Yunnan Province. Int J Climate Change Strateg Manage. (2023) 15:790–809. doi: 10.1108/IJCCSM-03-2023-0044

[33] Zhao C Si J Feng Q Luo H Qin J. Transformation of livelihood strategy for herdsman in Badain Jaran Desert and its impact on ecological environment. J Desert Res. (2020) 40:34–42. doi: 10.7522/j.issn.1000-694X.2020.00026

[34] Byrne T Chassler D Tamta M Muroff J Anderson R BenYishay M . Assessing anxiety and depression trajectories among single homeless adults receiving rapid rehousing following placement in housing. Housing Stud. (2025) 40:1944–66. doi: 10.1080/02673037.2024.2386280

[35] Zhao C Tang M Wang C. The impact of livelihood capital on the social integration of relocated households: mediating effects based on livelihood risk. Front Sustain Food Syst. (2025) 9:1537141. doi: 10.3389/FSUFS.2025.1537141

[36] Wang C Hao J Solomon T Liu H Liu D He Y. The impact of livelihood capital on farmers’ willingness to participate in wildlife conservation: evidence from the communities around the Jiyuan macaque nature Reserve in China. Sustainability. (2025) 17:7332. doi: 10.3390/su17167332

